# Digging deeper: exploring chiropractors online claims about non-musculoskeletal disorders

**DOI:** 10.1186/s12998-021-00407-z

**Published:** 2021-12-06

**Authors:** Søren O’Neill, Anneline Bugge Wahlqvist, Natasja Kragh Simonsen, Cornelius Myburgh, Rikke Krüger Jensen

**Affiliations:** 1 Spinecenter of Southern Denmark, University Hospital of Southern Denmark, Østre Hougvej 55, 5500 Middelfart, Denmark; 2 Institute of Regional Health Research, University of Southern Denmark, Odense, Denmark; 3 Department of Sports Science and Clinical Biomechanics, University of Southern Denmark, Odense, Denmark; 4 Chiropractic Knowledge Hub, University of Southern Denmark, Odense, Denmark

**Keywords:** Chiropractic, Web page, Non-musculoskeletal, Scope of practice, Subluxation, Type-O

## Abstract

**Background:**

Some chiropractors suggest that chiropractic treatment is appropriate for health issues other than musculoskeletal problems. The prevalence of such claims on individual clinic websites has previously been reported as approximately one-in-four in Denmark. The underlying rationales for such claims may reflect convictions about traditional chiropractic subluxations paradigms, but are not self-evident and has not previously been studied.

**Methods:**

An exploratory qualitative case interview study of Danish chiropractors with websites which contain claims about chiropractic efficacy in the treatment of non-musculoskeletal disorders. Websites were identified from a nation wide random sample (57%) of all chiropractic clinic websites.

**Results:**

Of the original 139 websites, 36 were identified as mentioning non-MSK conditions. When revisited, 19 of those clinic websites still mentioned non-MSK disorders and were contacted. Eleven (11) declined our invitation to participate. Interviews were conducted with the responsible chiropractor from each of the remaining 8 clinics.

Five distinct themes were identified in the rationales for treating non-musculoskeletal disorders: ‘Positive side-effects,’ ‘Experience,’ ‘Web page,’ ‘Communication’ and ‘Conviction.’

**Conclusions:**

A minority of Danish chiropractic websites suggest that non-musculoskeletal disorders are within the chiropractic scope of practice. Those that do, do so for varying reasons—poor communication and website maintenance were commonly cited problems. An explicitly stated adherence to traditional chiropractic subluxations concepts was uncommon. By contrast, a more tempered rationale that suggested a potential beneficial *side-effect* of chiropractic on non-musculoskeletal health issues were more common and was typically presented in softer-language and/or with some reservations.

**Supplementary Information:**

The online version contains supplementary material available at 10.1186/s12998-021-00407-z.

## Introduction

The heterogeneity of the chiropractic profession includes some chiropractors who pursue *mainstream integration*, promoting a diagnosis driven, *evidence based practice* approach strictly limited to *musculoskeletal* (MSK) problems. Other chiropractors promote a *chiropractic lifestyle* with regular spinal *adjusting* of *subluxations* to treat and prevent disease in general, including non-MSK problems (also known as type-O disorders) [1–3].

A recent comprehensive review of the literature concluded that there is only limited evidence, but it indicates that spinal manipulation is not effective for the management of non-MSK disorders [4]. Additionally, in a recent commentary on chiropractic professional heterogeneity, LeBoeuf-Yde et al. [5] observed that “Today, the division is between the ‘evidence-friendly’ faction that focuses on musculoskeletal problems based on a contemporary and evidence-based paradigm, and the ‘traditional’ group that subscribes to concepts such as ‘subluxation’”. The readiness of chiropractors to treat non-MSK conditions could thus be interpreted as an indicator of their position on a professional spectrum from the traditional subluxation paradigm to the modern evidence based approach.

In a previous study, Jensen et al. examined the websites of a random sample of Danish chiropractors for any mention of non-MSK problems [6]. Twenty-six percent (26%) of the sampled websites mentioned such non-MSK diagnoses or symptoms. Similar observations have been reported elsewhere, with approximately 30% of sampled chiropractic websites in Canada mentioning asthma or allergies [7] or attention deficit disorder, pre-menstrual syndrome, allergies or bed-wetting [2].

When chiropractors advertise treatment for non-MSK disorder, one plausible interpretation could thus be, that it is indicative of an adherence to ‘traditional’ chiropractic dogmas about subluxations, vitalism, innate intelligence, etc. It is not the only possible explanation however, and several others come to mind: (i) MSK problems can negatively impact non-MSK problems and vice versa. E.g. obesity and diabetes negatively impacts, and is negatively impacted by the reduced physical activity which often accompanies painful MSK disorders. (ii) Misdiagnosis of MSK problems as non-MSK problems and vice versa. E.g. attributing chest pain to angina pectoris, when in fact it stems from MSK structures. (iii) *Chiropractic treatment* is often used synonymously with spinal manipulation, but may in fact encompass advice on nutrition, exercise, pain medication, etc. All of which may have direct bearing on some non-MSK health issues.

Explanations such as (i)–(iii) above may be important in the management of individual patients and could possibly lead to mentioning of non-MSK disorders on websites. If that is indeed the case, it underlines the importance of clear and unambiguous communication—chiropractors are not the appropriate first contact for suspected coronary disease or diabetes and public communications should take care not to mislead patients in such regard.

It thus questions whether the mentioning of non-MSK disorders on chiropractic websites can be equated with an adherence to historical subluxation dogmas. Exploring the underlying rationales for mentioning non-MSK disorders more deeply, might reveal other explanations such as those suggested above.

The present study aimed to examine in greater detail, the underlying reasons or rationales for mentioning non-MSK disorders on Danish chiropractors websites.

## Methods

### Study design

An exploratory qualitative case study.

### Investigator group

The investigator group consisted of three senior researchers and two final year chiropractic students. All three senior researchers (SON, CM, RKJ) have backgrounds as licensed chiropractors and have past experience from private clinical chiropractic practice, albeit not so for several years. One (SON) continues to hold part-time clinical employment in a regional hospital. The two students (NSK, ABW) were involved in the project as part of their master theses studies.

None of the investigators had prior contact or relations with the study participants.

### Population and sampling strategy

**Fig. 1:**
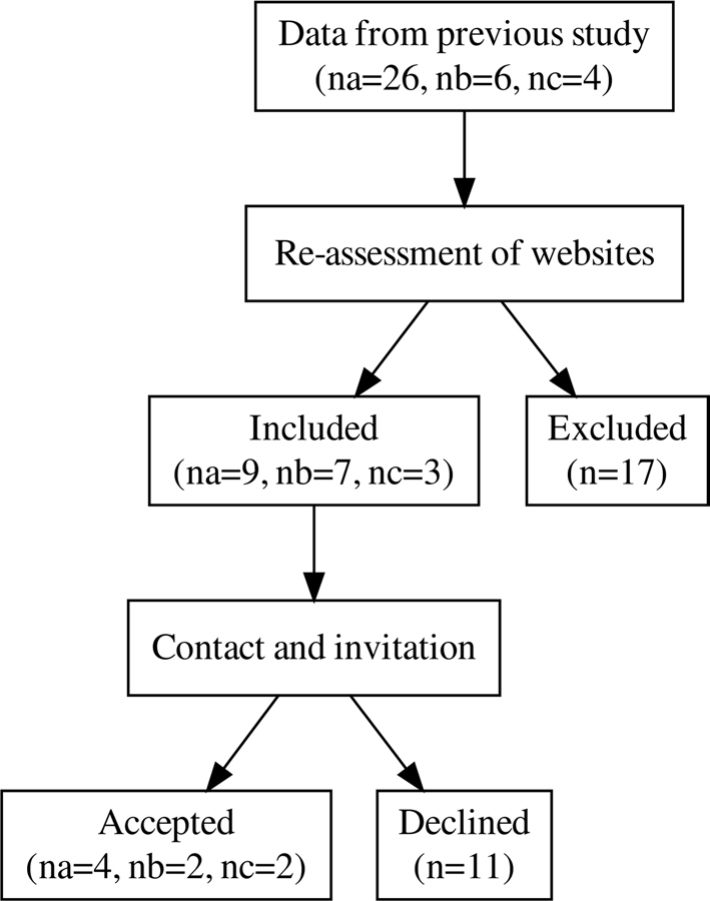
Flow diagram of study structure (na, nb and nc refers to numbers in category a, b and c, defined as *1–2*, *3–5* and *6 or more* non-MSK conditions mentioned on webpage)

In order to identify relevant websites we used data from a previous study examining the prevalence of mentioning non-MSK health issues on Danish chiropractic websites [6]. That study was based on a geographically stratified random sample (n = 139, 57%) of all Danish chiropractic clinic websites of which 36 mentioned non-MSK disorders. As that data had been collected in 2019 those clinics’ websites were re-visited and their categorizations were re-evaluated to examined whether they still mentioned one or more non-MSK conditions. In cases where a clinic website no longer mentioned non-MSK diagnoses or symptoms, they were excluded (see Fig. 1).

Thus a total of 19 eligible websites from a representative national sample were identified as mentioning non-MSK conditions. Using a criterion approach, the websites were categorized on the basis of the number of different non-MSK conditions mentioned, as (a) 1–2 conditions, (b) 3–5 conditions and (c) 6 or more conditions.

We aimed to include four inclusion-eligible clinics from each category, chosen at random from a pre-generated randomization list. Categorization was based on the assumption that a high number of conditions mentioned could reflect a different view on non-MSK conditions compared to if only a few conditions were mentioned.

### Recruitment

The randomly chosen inclusion-eligible clinics were contacted by NKS and ABW: Clinics operated by a single chiropractor were contacted directly by email and invited to take part in the study (see Appendix 1 and 2 in Additional file 1). For clinics with multiple chiropractors, a clinic secretary was contacted and asked to pass the invitation on to the chiropractor responsible for the clinic website content.

If a clinic failed to respond within 4 days, the clinic was contacted again and encouraged to respond to the invitation, either way.

In those instances where a clinic declined to participate, a replacement from the same category was chosen randomly from the a fore mentioned randomization list. If a category was exhausted before including four clinics at random, a replacement was chosen randomly from the larger of the other categories.

### Interviews and data collection

Interviews were conducted between September 1st and 25th (2020) by NKS and ABW. The interviewers and interviewees had no prior contacts or relations. No pilot interviews were conducted.

Due to the Covid-19 pandemic, interviews took place via an online video conference platform (Zoom v5.0.2 (24030.0508), Zoom Video Communications, San Jose, CAL, USA) and, with the participating chiropractors’ consent, all interviews were recorded digitally for later analyses (sound track only). This replaced interview field notes.

During the interview, the participating chiropractors were asked to elaborate on the thinking behind their own clinic websites mention of non-MSK disorder.

Furthermore, a prepared vignette consisting of three text segments were used to facilitate the interview and provide cues for the chiropractor to describe his/her thoughts on chiropractic treatment of non-MSK disorders (see Box 1). Vignettes were used as they allow for bracketing the topic of non-MSK disorders without affecting individual opinion [8]. The vignettes were randomly selected passages about non-MSK disorders from Danish chiropractic websites. The same vignettes were used in all interviews.

**Box 1:** Vignette consisting of three text segments used to facilitate the interviews

*The following descriptions of the chiropractic scope of practice have been seen on Danish chiropractors’ web pages:*
1. The relationship of the nervous system to the spinal column, is the reason why we can help patients with headache, high blood pressure, low blood pressure, reduce lung capacity, reduced immune capacity, stomach problems, etc. Conditions which can all be caused by interference of the communication between the brain and the organ
2. The children I treat, come to me because of problems crawling or walking, falling over a lot, problems sleeping, stomach pains or congestion, bed wetting, poor posture, foot imbalance, hyperactivity, restlessness, attention problems or ADHD-like problems
3. We also treat, muscle infiltrations, dizziness, whiplash injuries, skull and jaw problems, middle ear problems, shoulder and arm pain, tennis and golfers elbows, carpal tunnel syndrome, groin pain, hip pain, knee problems, foot problems, ankle problems, indigestion. All of the above and much more, we can help you with
*What do you think, when you read these examples?*

Interviews were transcribed verbatim and data were collated and organized using NVivo12 (QSR international, MAC version 12.3.0 (3508)). The content was analyzed as per the ‘Systematic Text Condensation’ methods [9] guided by ‘A hands-on guide to doing content analysis’ [10]. No repeat- or follow-up interviews were performed.

### Data analyses

The qualitative analysis method was systematic text condensation which is appropriate for qualitative cross-case analysis, and manageable for inexperienced researchers as it requires no grounding in a particular qualitative research tradition [9]. Furthermore, it allows for intersubjectivity, reflexivity, and feasibility. As a method for data generation un-structured (or non-directive) interviews of eligible respondents identified from a stratified, random sample of Danish chiropractors were chosen in order to explore explanations and opinions of the website owners as well as understand the motivation and discover perspectives of interest.

**Fig. 2:**
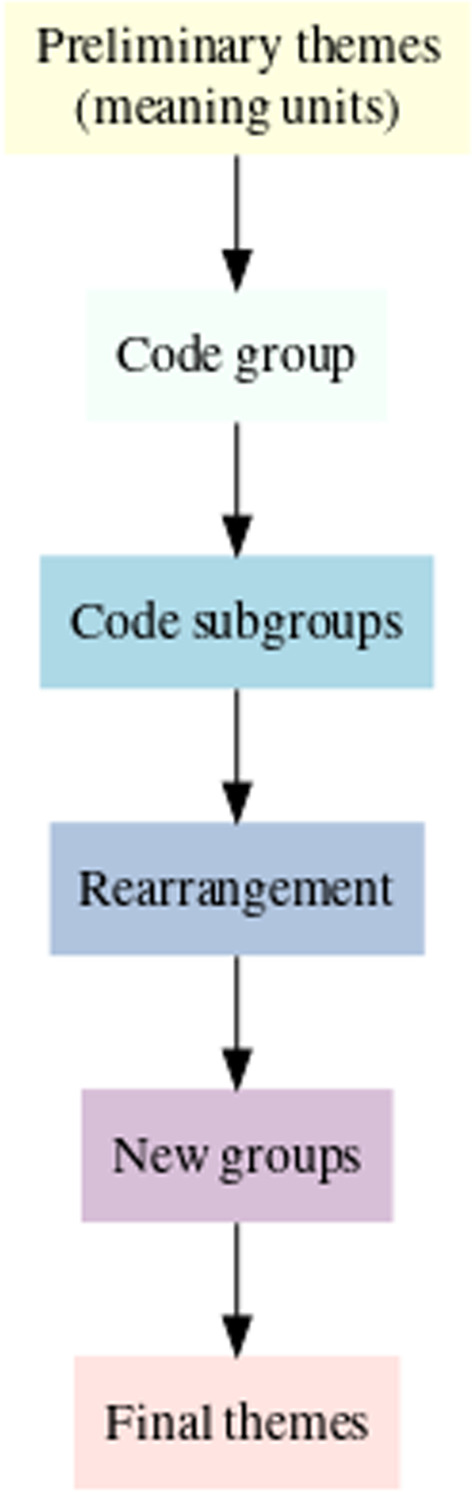
Flow diagram of data analysis

The interview content themes were identified by NKS and ABW through a structured, multi-step process (see Fig. 2).

Each transcribed interview was read and re-read repeatedly by both analysts independently until a list of *meaning units* had been identified and no further meaning units were found on further readings. The two preliminary lists of meaning units were then compared and discussed by the analysts in relation to the study aims and any redundant or irrelevant meaning units were deleted.

The remaining meaning units were re-assessed with the aim of collating them into larger *code groups* which could encompass one or more themes.

Each code group was subdivided into subgroups representing different perspectives on the code groups.

The analysts re-visited the filtered list of meaning units to determine how they related to the code groups and their subgroups. Subgroups were rearranged, split and/or merged to ensure that all meaning units were encompassed in a code group and subgroup. Raw data (interview statements) representative of the subgroups were identified and code groups were given descriptive titles. These descriptive titles and illustrative interview statements together formed the final result of the analyses, i.e. recurring rationales for mentioning non-MSK disorders on chiropractic websites.

### Ethics

Data was collected anonymously with no person- or clinic identifiers and was stored in accordance with the European GDPR regulations. Informed consent was obtained from all participants prior to data collection. Danish research ethics committees need not be notified of surveys using questionnaires and interviews that do not involve human biological material (Sect. 14(2) of the Committee Act) [11].

## Results

Eight interviews were conducted in total between September 1st and 25th (2020) by NKS (n = 4) and ABW (n = 4).

### Population sample

The data set from Jensen et al. [6] included 26 websites in category (a), 6 in (b) and 4 websites in (c). After randomization and iteratively revisiting and re-evaluation the websites, it was found that 17 of these had been amended and no longer fulfilled the inclusion criteria. Of the remaining 19 clinic websites, 9 were categorized as (a), 7 as (b) and 3 as (c).

Eleven of the inclusion eligible clinics declined our invitation to take part in the study, leaving a total of 8 participating clinics—4 in category (a) and 2 in each of (b) and (c).

The specific non-MSK health issues mentioned on the websites of participating clinics are listed in Table 1.

**Table 1:** Specific non-MSK disorders mentioned on websites

Non-MSK disorders
Allergies
Asthma
Digestive problems
Mood disorders
Hyperactivity/restlessness
High/low blood pressure
Immune responsiveness
Incontinence/bed wetting
Attention deficits
Middle ear infections
Decreased lung capacity
Vestibular neuritis
Language, reading and writing difficulties
Insomnia/unease

### Thematic analysis

Initially, eight themes were identified: ‘Experience,’ ‘Web page,’ ‘Communication,’ ‘Nervous system,’ ‘Conviction,’ ‘Patient expectation’ and ‘Training.’

**Fig. 3:**
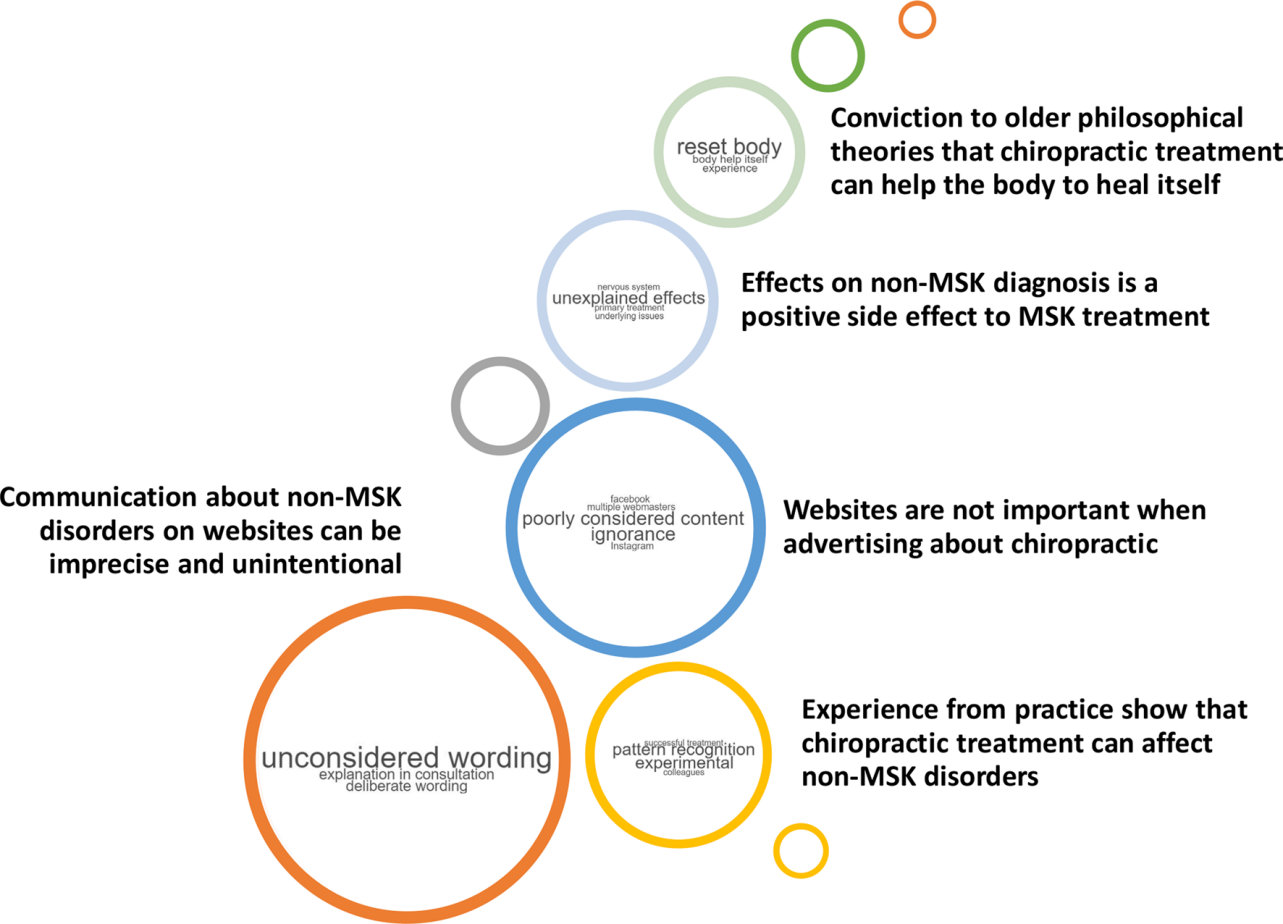
Word cloud presentation of final themes and their meaning

After analyses, coding, grouping and re-arranging, five final themes had been identified: ‘Positive side-effects,’ ‘Experience,’ ‘Web page,’ ‘Communication’ and ‘Conviction.’ Illustrative raw-data quotes from each theme are listed in Appendix 3 in Additional file 1. The five final themes are presented below in a word cloud—Fig. 3.

### Positive side-effect—Effects on non-MSK diagnosis is a positive side effect to MSK treatment

The first theme explained how chiropractors regarded treatment effects on non-MSK disorders as positive side-effects when treating MSK disorders.

There was broad agreement among the interviewed chiropractors that a chiropractor’s focus area is the musculoskeletal system:

So, if we’re to make a difference, we actually need to deal with the musculoskeletal system - full stop. [I1]

However, several chiropractors mentioned observing positive side effects on non-MSK disorders when treating MSK disorders. Some expressed a presumed link between the positive side effect and the nervous system, but the general attitude was that the positive side effect was unexplained.

But sometimes you’re lucky that something happens which has an effect, and we can’t always explain why. [I3]

The interviewed chiropractors sometimes informed patients about the possibility of treatment effects on non-MSK disorders. If so, they emphasized that it was a prerequisite to have an in-depth conversation with the patients before or during the consultation, where they explained the relationship between the positive side effect and underlying musculoskeletal problems.

..so if I treat it, I’ll tell the patient – or rather the parents more commonly – that actually that’s not really what I’m treating. I treat what I find in the child. So, I always treat musculoskeletal, always the spine, but it might in turn help the bed wetting. [I6]

### Experience—clinical experience that chiropractic treatment is relevant for non-MSK disorders

The second theme suggested that many years of clinical experience had convincingly demonstrated that chiropractic treatment was relevant for non-MSK disorders. The interviewed chiropractors could not explain *how* treatment was effective and were aware that this was not mainstream. They explained however, that they had been in the profession for so long that they had acquired the experience to distinguish the patients with non-MSK disorders that would benefit from chiropractic treatment and those who would not.

It’s just that as a clinician when you get experience dealing with a group, e.g. children and you’ve been doing it for years, you see a thread in who you can help, and you can also quickly dismiss those you see you can’t. [I8] It’s quite simply, just experience. And we write these things on the webpage, because I don’t care anymore. I’ve been doing this for a long time. [I7]

The clinical experience of older colleagues was held in high regard and inexperienced chiropractors with an open mind would rely on the experience of such colleges and rely on that.

The first few years I relied on my colleague’s cases and presentations, etc. I might have said, ‘I know colleagues have been successful with this and I’d like to give it a try.’ Again, over the years it’s been my experience – by lots of experience and keeping your eyes open and remaining curious – you actually see quite a lot [I1]

Knowledge about treatment possibilities of non-MSK disorders in children apparently also came from courses although the communication about it seemed vague.

It’s because we have attended different courses – baby-courses where we have been given … what can you say, information.. [I5]

### Webpage—websites are not important when advertising chiropractic

The third theme encompass the view that websites were not considered important in the communication with patients and were therefore not well maintained.

Many of the chiropractors stated that they did not really know what was on their website, and therefore could not explain why non-MSK disorders were mentioned. Social media had in some cases overtaken websites as primary platform of communication with patients.

We’re probably better at using Facebook and Instagram which we update on a weekly basis, and the webpage is kind of forgotten. Of course, it shouldn’t be, when it’s there. [I8]

Interviewees reported there was no real incentive to maintain websites with regard to competition as personal recommendation from one patient to another was considered more important than advertisement on websites.

We don’t have a lot of competition on our website either. In [town] we are three chiropractic clinics, so when someone searches ‘Chiropractor in [town]’ our link always comes up. It’s different if you search for ‘Carpenter Copenhagen’ where you’ll get 40 or 50 or however many, then there’s competition about who is what on the webpage [I3] Yeah. Well, nine out of ten that come to see us come because, ‘Well, someone in my family said you’re really good’ or ‘My neighbour told me to see you, because you can work it out.’ That’s how most people come here and that’s why I think we’re not particularly motivated to do a whole about our webpage, because it’s not that important [I3]

In clinics with more than one owner, one did not always know what the others had written on the website and it was evident that many of the chiropractors did not regularly look at their own website. One of the chiropractors mentioned that the website was last revised 10 years ago.

..I’d actually forgotten, that I wrote that. It’s been a long time, since I made the webpage and you don’t visit it that often, at least I don’t [I6]

Shared clinic ownership also meant that some website content represented a compromise of convictions.

And I’m not the only one who defines the clinic – you have to remember, that I have a colleague – and he’s a bit more alternative than I am, so sometimes it’s a compromise. [I3]

### Communication—wording about non-MSK disorders on websites can be imprecise and unintentional

Several chiropractors stated that it was just a case of imprecise wording which did not reflect the actual intention. They were not aware that the wording could be interpreted in a different way than intended.

No, I probably ought to actually. I mean, it should be clearer that it is still the spine that I’m treating, as it were, or whatever I find. Yes, you’re right, I probably should write that it’s a kind of side-effect. [I6] I can follow what you’re saying, perhaps it should be removed or simply write that viral infection of the balance nerve, is a dizziness but not one that you can be treated for here at our clinic. I never thought about that before, or had it pointed out. [I4]

### Conviction—adherence to older philosophical theories that chiropractic treatment can help the body to heal itself

Only one chiropractor was practicing according to older chiropractic theories. The chiropractors view was the basic idea that spinal manipulation can stimulate the body to heal itself of both MSK and non-MSK conditions.

If I can reset the skeleton and your body in some way – help your body to help itself. Because chiropractic doesn’t heal anything, not even doctors heal anything. It’s only the body that heals itself, as far as I know. [I7]

## Discussion

The current study examined the reasons and reasoning behind the minority of Danish chiropractic websites’ which mention non-musculoskeletal disorders. We identified five distinct themes, which ranged from the practicalities of maintaining a website to particular convictions about the scope of chiropractic practice.

Of the five themes identified in the current study, ‘Conviction’ was only really expressed by a single chiropractor. This suggests to us, that an actual and *explicitly expressed* chiropractic scope of practice which includes non-MSK disorders as a clinical indication for chiropractic care, is uncommon in a Danish context. However, we can not exclude that our data is biased in this regard as eleven clinics declined our invitation to participate—it is entirely possible, that clinics with such a scope of practice would systematically decline participation. In fact, revisiting those websites (post-hoc) confirmed that all but one still listed such conditions as indicators for chiropractic care. We can not assess the underlying reasoning, however.

Conversely, we also observed that seventeen (17) clinics previously found by Jensen et al. [6] to mention non-MSK disorders had amended their websites and were no longer eligible for inclusion. It is possible that those amendments reflect a desire to *explicitly express* a scope of practice which does not included non-MSK disorders. It should be noted, that following the publication of the paper by Jensen et al. [6], the Danish Chiropractic Association campaigned to make Danish chiropractors aware of the topic and the controversies surrounding it. At face value then, a practice paradigm which includes non-MSK disorders appears to be a relatively uncommon professional stance in our context.

This finding contrasts with previous reports from the United States [12], that 75% of surveyed chiropractors favored a *broad scope* of practice and empirically found that spinal manipulation of vertebral *subluxations* “usually elicits improvements in select visceral ailments” (non-MSK disorders). Australian and Canadian surveys report that *subluxation* was mentioned on 28% and 33% of chiropractic websites respectively [3, 13], i.e. similar percentages to the websites that make mention of non-MSK disorders [2, 7]. Based on our results, the mentioning of non-MSK disorders in a Danish context does not seem to equate to an adherence to concepts such as a subluxation based practice paradigm but could have several explanations, discussed in the following paragraphs.

The themes ‘Web page’ and ‘Communication’ reflect aspects of communication. In some cases, chiropractors reported that social media platforms were prioritized at the expense of clinic websites which therefor grew stale and outdated—e.g. “We’re probably better at using Facebook and Instagram which we update on a weekly basis, and the webpage is kinda forgotten.” In other cases, the website was simply considered to be of little consequence and more or less ignored. In any case, statements in these themes did not necessarily make clear the reasons why non-MSK disorders were mentioned on the websites in the first place. Some explained that the website wording represented a compromise between different chiropractors with different perspectives on the treatment of non-MSK disorders. Arguably, some of those chiropractors would align more with the ‘Conviction’ theme if not required to compromise with their colleagues, but by the same token some chiropractors, if not having to compromise with their colleagues might not have made mention of non-MSK disorders at all.

A more common rationale expressed in our data, captured by the themes ‘Positive side-effect’ and ‘Experience’ was that of a positive but *unexplained* treatment outcome on non-MSK disorders. The central issue here seemed to be one of two rationales: (i) treatment was directed at an MSK disorder and any positive effect on non-MSK health issues was considered a spontaneous, serendipitous bonus—e.g. “But sometime you’re lucky that something happens which has an effect, and we can’t always explain why.”, or (ii) in fact the chiropractor had an expectation that treatment of MSK disorders would positively impact non-MSK disorders, but stressed that the MSK disorder was the object of treatment—e.g. “So, I always treat musculoskeletal, always the spine, but it might in turn help the bed wetting.”

At first reading, this seems to imply that the chiropractor *does not* offer treatment for non-MSK problems, but it begs the question how these themes and underlying rationales are fundamentally different from the ‘Conviction’ theme. The language and specific explanatory model of a subluxation based paradigm is avoided, but the central tenet remains the same: That a causal link exists between MSK disorders which are amendable to spinal manipulation and non-MSK health problems. Describing the underlying mechanism as ‘unexplained’ or the clinical outcome as ‘uncertain but worth a try’ does not fundamentally change that. In fact it hints at an underlying acceptance of the core of the traditional chiropractic theories, namely that of the vertebral *subluxation* (by whatever name) as a potentially significant cause of non-MSK health issues. It could therefor be argued, that the rationale behind these two themes is essentially a variant of the ‘Conviction’ theme albeit modernized and adapted without the controversial baggage of a subluxation paradigm. As such, perhaps unwittingly, the proverbial subluxation-baby was indeed thrown out with the bath water but was then sneaked back in again through the basement.

In summary then, since the first survey [6] seventeen of thirty-six clinic websites had been amended in such a way as to no longer make mention of non-MSK disorders, and of the websites which still did, some indicated that this was due to websites being outdated and poorly maintained. It also seems clear however, that a number of clinics still advertise chiropractic care as relevant for at least some non-MSK disorder, albeit using softer language and hedging claims about clinical efficacy.

A case can be made, that in fact the question about chiropractic management of non-MSK disorders ought to be of little interest: McDonald [12] reported that three-quarters of American chiropractors favored a broad scope of practice including non-MSK disorders, but in actuality three-quarters of patients consult chiropractors with low back and neck pain [14] and the rest is made up mostly of other MSK problems such as extremity joint pain, thoracic pain, tension type headache, etc. In North America, non-MSK disorders are given as the reason for consulting a chiropractor in less than 7% of cases [15, 16], in Belgium it is less than 3% [17] and in the Netherlands less than 2% [18]. Within a Danish context, we suspect the public perception of the relevance of chiropractic unambiguously tied to the management of musculoskeletal problems and not non-MSK health issues, in alignment with findings in other countries [19, 20]. We venture to suggest, that the medical profession, public health authorities and third-party payers are unlikely to see it differently [21, 22]. In other words and in very real numbers, non-MSK health issues constitutes a negligible proportion of actual chiropractic practice and the perception of chiropractic as relevant for non-MSK disorders, seems confined within the profession itself. By contrast, pushing chiropractic as relevant for non-MSK disorders will likely muddled the professions’ public image and stimulate skepticism or outright disregard by scientific, political and professional communities—and that by contrast, ought to be of considerable interest to the profession.

## Conclusion

This study demonstrated that the underlying reasons or rationales for mentioning non-MSK disorders on a minority of Danish chiropractic websites were not a simple reflection of a traditional subluxation paradigm. Poor maintenance of websites, unclear phrasing and a tempered expectation that treatment might help and certainly not do harm were uncovered as common reasons.

## Supplementary Information

**Additional file 1.** Invitation email and additional information, and illustrative quotes of identified themes.

## Data Availability

The data used and/or analyzed during the current study are available from the corresponding author on reasonable request.
