# Supplementary material for: Digging deeper: exploring chiropractors online claims about non-musculoskeletal disorders
Source: Chiropr Man Therap. 2021 Dec 6;29:50. doi: 10.1186/s12998-021-00407-z (PMC8646014; doi:10.1186/s12998-021-00407-z)
Supplement: Supplementary file 1 — Additional file 1. Invitation email and additional information, and illustrative quotes of identified themes. [file 12998_2021_407_MOESM1_ESM.docx]

Appendices

Digging deeper: exploring chiropractors online claims about non-musculoskeletal disorders

# Appendix 1

## Participant email

“Did you know, that 10% of Danish chiropractors’ websites mention attention deficit problems in children? And that 26% mention one or more types of non-musculoskeletal health issues on their webpages?”

We are curious about what this means and would like to understand, what actually lies behind the numbers.

We invite you to take part in an interview, as your webpage mentions some such examples on non-musculoskeletal issues.

Your interview will be part of a scientific publication which will examine the meaning and rationale for mentioning non-musculoskeletal issues on chiropractic websites.

We will take the liberty of contacting you within the next couple of days to make arrangements for your participation.

More information is provided in the attached materials.

Kind regards Natasja Kragh Simonsen and Anneline Bugge Wahlqvist

# Appendix 2

## Additional information

Date and time of interview is arranged

The interview will be condiucted via the virtuelle platform ZOOM and is expected to last about 30 minutes.

With your consent, we will record the interview, so that the audio recording can be used for data analyses.

All personal information and statements in connection with the study will be anonymized and secured in accordance with current guidelines.

By taking part in the study you consent to the anonymized data being used in the thesis and subsequent research.

You can, at any time withdraw your consent.

Contact information:

NSK – email

ABW – email

# Appendix 3

## Illustrative quotes by final themes

### ‘A positive side-effect’

“So if we’re to make a difference, we actually need to deal with the musculoskeletal system - full stop.” [I1]

“I hope what we write is, that we treat fixations in the neck and that we see as a positive side-effect that sometimes, middle ear fluid cleares up, if it caused by tension.” [I2]

“But sometime you’re lucky that something happens which has an effect, and we can’t always explain why.” [I3]

“..so if I treat it, I’ll tell the patient – or rather the parents more commonly – that actually that’s not really what I’m treating. I treat what I find in the child. So, I always treat musculoskeletal, always the spine, but it might in turn help the bedwetting.” [I6]

### Experience

“It’s just that as a clinician when you get experience dealing with a group, e.g. children and you’ve been doing it for years, you see a thread in who you can help, and you can also quickly dismiss those you see you can’t.” [I8]

“It’s quite simply, just experience. And we write these things on the webpage, because I don’t care anymore. I’ve been doing this for a long time.” [I7]

“The first few years I relied on my collegaues cases and presentations, etc. I might have said, ‘I know collegaues have been successful with this and I’d like to give it a try.’. Again, over the years it’s been my experience – by lots of experience and keeping your eyes open and remaining curious – you actually see quite a lot” [I1]

“It’s because we have attended different courses – baby-courses where we have been given … what can you say, information..” [I5]

### Webpage

“We’re probably better at using Facebook and Instagram which we update on a weekly basis, and the webpage is kinda forgotten. Of course it shouldn’t been, when it’s there.” [I8]

“We don’t have a lot of competition on our website either. In [town] we are three chiropractic clinics, so when someone searhes ‘Chiropractor in [town]’ our link always comes up. It’s different if you search for ‘Carpenter Copenhagen’ where you’ll get 40 or 50 or how ever many, then there’s competition about who is what on the webpage” [I3]

“Yeah. Well, nine out of ten that come to see us come because, ‘Well, someone in my family said you’re really good’ or ‘My neighbor told me to see you, because you can work it out’. That’s how most people come here and that’s why I think we’re not particularly motivated to do a whole about our webpage, because it’s not that important” [I3]

“And I’m not the only one who defines the clinic – you have to remember, that I have a colleague – and he’s a bit more alternative than I am, so sometimes it’s a compromise.” [I3]

“..I’d actually forgotten, that I wrote that. It’s been a long time, since I made the webpage and you don’t visit it that ofte, at least I don’t” [I6]

### Communication

“But of course, its about attracting the right group without, well you don’t want to frighten people away, but on the other side we don’t want to attract all the kids with attention problems because we can’t help them. It’s about getting the right group.”[I8]

“No, I probably ought to actually. I mean, it should be more clear that it is still the spine that I’m treating, as it were, or what ever I find. Yes, you’re right, I probably should write that it’s a kind of side-effect.” [I6]

“I can follow what you’re saying, perhaps it should be removed eller simply write that viral infection of the balance nerve, is a dizziness but not one that you can be treated for here at our clinic. I never thought about that before, or had it pointed out.” [I4]

“.. it’s no longer a question of conviction and I can tell from the patients. Instead of having to tell them this and that, they already understand. They’ve heard and read about it and understand it. I don’t have to convince people, because they already know what we actually do and not just something, that someone believes in.” [I2]

### Conviction

“If I can reset the skeleton and your body in some way – help your body to help it self. Because chiropractic doesn’t heal anything, not even doctors heal anything. It’s only the body hat heals itself, as fas as I know.” [I7]
